# The relationship of maternal and child methylation of the glucocorticoid receptor NR3C1 during early childhood and subsequent child psychopathology at school-age in the context of maternal interpersonal violence-related post-traumatic stress disorder

**DOI:** 10.3389/fpsyt.2022.919820

**Published:** 2022-08-19

**Authors:** María I. Cordero, Ludwig Stenz, Dominik A. Moser, Sandra Rusconi Serpa, Ariane Paoloni-Giacobino, Daniel Scott Schechter

**Affiliations:** ^1^Department of Psychology, Manchester Metropolitan University, Manchester, United Kingdom; ^2^Department of Genetic Medicine and Development, University of Geneva Faculty of Medicine, Geneva, Switzerland; ^3^Child and Adolescent Psychiatry Service, Lausanne University Hospital, Lausanne, Switzerland; ^4^Department of Psychology, University of Geneva Faculty of Psychology, Social Science and Education, Geneva, Switzerland; ^5^Department of Psychiatry, Lausanne University Faculty of Biology and Medicine, Lausanne, Switzerland; ^6^Department of Child and Adolescent Psychiatry, New York University Grossman School of Medicine, New York, NY, United States

**Keywords:** intergenerational transmission of aggression, maternal PTSD, glucocorticoid receptor, epigenetics, child psychopathology

## Abstract

**Introduction:**

Interpersonal violent (IPV) experiences when they begin in childhood and continue in various forms during adulthood often lead to chronic post-traumatic stress disorder (PTSD) that is associated in multiple studies with hypocortisolism and lower percentage of methylation of the promoter region of the gene coding for the glucocorticoid receptor (NR3C1). This prospective, longitudinal study examined the relationship of NR3C1 methylation among mothers with IPV-related PTSD and their toddlers and then looked at the relationship of maternal NR3C1 methylation and child psychopathology at school age.

**Methods:**

Forty-eight mothers were evaluated for life-events history and post-traumatic stress disorder *via* structured clinical interview when their children were ages 12–42 months (mean age 26.7 months, SD 8.8). Their children's psychopathology in terms of internalizing symptoms and externalizing behaviors was evaluated using the Child Behavior Checklist at ages 5–9 years (mean age 7 years, SD 1.1). Percentage of methylation for the NR3C1 gene promoter region was assessed from DNA extracted from maternal and child saliva using bisulfite pyrosequencing. Data analysis involved parametric and non-parametric correlations and multiple linear and logistic regression modeling.

**Results:**

Logistic regression models using child NR3C1 methylation as the dependent variable and maternal NR3C1 methylation and PTSD group status as predictors, as well as the interaction indicated that all three of these significantly predicted child NR3C1 methylation. These findings remained significant when controlling for child age, sex and maternal child abuse history. Overall, maternal NR3C1 methylation when children were toddlers was negatively and significantly associated with child externalizing behavior severity at school age.

**Discussion:**

We found that correlations between mothers and their children of NR3C1 methylation levels overall and at all individual CpG sites of interest were significant only in the IPV-PTSD group. The latter findings support that NR3C1 methylation in mothers positively and statistically significantly correlates with NR3C1 methylation in their children only in presence of IPV-PTSD in the mothers. This maternal epigenetic signature with respect to this glucocorticoid receptor is significantly associated with child behavior that may well pose a risk for intergenerational transmission of violence and related psychopathology.

## Introduction

The nuclear receptor subfamily 3, group C, member 1 (NR3C1) gene encodes the glucocorticoid receptor (GR) involved in the hypothalamic–pituitary–adrenal axis (HPA). The NR3C1 exon 1_7_ in rats and 1_F_ in humans behave as a promoter whose methylation levels are important for response to stress (1). One of the most extreme forms of stress at least among humans is that of a threat to one's life and/or bodily integrity such as during the experience or witnessing of violent physical, sexual or otherwise interpersonal violence (2, 3). This type of traumatic event(s) is particularly pathogenic for the development of posttraumatic stress disorder (PTSD), a psychiatric illness affecting on average 10.4% of women in community samples (4), but can affect 51–75% of women who are victims of domestic violence depending on the measures used to evaluate PTSD (5, 6). PTSD is characterized by a combination of re-experiencing symptoms, avoidance of traumatic reminders—at great cost of psychic energy, as well as hyperarousal symptoms and negative cognitions involving anticipation of a foreclosed future, feelings of guilt and even suicidal ideation (7, 8). Previous published studies have demonstrated the relationship of the posttraumatic stress disorder and decreased methylation of the NR3C1 promoter region (9–11). Furthermore, Yehuda et al. had promising findings (though with a very small sample of combat veterans with PTSD) that suggest that DNA methylation of NR3C1 exon 1F could predict the outcome of a psychotherapy-based treatment (12).

Decreased methylation of the NR3C1 gene promoter region has, in turn, been associated with lower peripheral glucocorticoid levels and reduced reactivity to stressful laboratory stimuli (13).

The first direct demonstration of NR3C1 exon 1_F_ methylation alterations in relation to parental PTSD was reported in the context of Holocaust survivors; the presence of both maternal and paternal PTSD resulted in lower methylation of NR3C1 exon 1_F_ in the offspring (14). Despite this finding, while exposure to trauma and subsequent PTSD has often been linked to hypomethylation of NR3C1 exon 1_F_ in adults, yet with increased methylation of NR3C1 in children exposed to traumatic events. Recently, a systematic review conducted on child maltreatment reported that methylation in exon 1_F_ of NR3C1 increased in presence of child maltreatment as well as in children exposed to intimate partner violence in the majority of the studies (11).

We previously assessed NR3C1 methylation in mothers with Interpersonal violence-related posttraumatic stress disorder (IPV-PTSD), all of whom having a history of adult exposure to interpersonal violence and the vast majority of those, having experienced childhood maltreatment and family violence exposure (10). We found that maternal IPV-PTSD and its severity were associated with decreased methylation of the NR3C1 promoter region, which in turn, correlated with functional brain activity in the mothers involving decreased ventral medial prefrontal cortical activity and increased right hippocampal activity (10). In a study derived from the same sample examining salivary cortisol levels, we found that mothers with IPV-PTSD and their children– by the age of 12–42 months, as compared to mothers without PTSD, and their children showed lower salivary cortisol baseline levels, mothers in particular having altered circadian rhythms, and children, lower cortisol reactivity to separation stress (14).

The goal of the present study involving mothers and children was to take our previous study one step further, by considering the relationship of maternal and child methylation of the NR3C1 promoter regions during an early developmental period sensitive to the caregiving environment that is adversely affected by maternal IPV-PTSD and its perturbation of psychobiological regulation of emotion and arousal. We then went on to look at the relationship between this early NR3C1 methylation signature, in terms of overall methylation and that of specific CpG islands as reported in our previous study (10, 15) and child behavior 4-years later during school age (5–9 years). One stimulus to write this paper was the publication of a recent study that investigated epigenetic covariance in mother-child dyads' degrees of methylation among four stress-regulation related genes (5HTT, NR3C1, FKBP5, and BDNF) in a sample of 160 typical peri-pubertal youth (ages 8–16) and their mothers (16). Results showed that mother and offspring NR3C1 methylation signatures were significantly correlated in that latter study. Interestingly, in a previous study, Yehuda et al. (9) reported a significant positive correlation of methylation in another gene that also regulates glucocorticoid receptor activity (FK506 binding protein 5, FKBP5) between parents and their offspring, in a sample including Holocaust survivors (*n* = 32) and match controls (*n* = 8) and their adult offspring (Holocaust offspring, *n* = 22; control offspring, *n* = 9); the correlation was significant when considering the total sample or the Holocaust sample, but not for the control sample (17). Yehuda et al. (9) argued that this intergenerational epigenetic transmission in the offspring of highly traumatized individuals may increase vulnerability to psychopathology in the F1 generation.

Van Aswegen et al. also pointed to the importance of considering this maternal-child relationship in the context of problematic mother-child relationships (16). Indeed, in our previous study, we showed maternal methylation of the NR3C1 promoter region as negatively and significantly associated with parenting stress, which is a known marker of parent-child relationship disturbance and is predictive of both child internalizing (i.e., anxiety and depression) and externalizing problems (i.e., disruptive behavior disorder symptoms such as those of attention deficit hyperactivity, oppositional defiant and disruptive mood dysregulation disorder) (10, 18). Parenting stress is known to be increased both in the context of maternal history of maltreatment and other interpersonal violence exposure as well as in the context of psychopathology such as PTSD and depression (19–22). In turn, child internalizing and externalizing symptoms have been associated with maternal history of maltreatment and exposure to other interpersonal violence (23, 24).

With these associations in mind, we reviewed the literature to see whether methylation of the maternal NR3C1 gene promoter region had been studied in relation to child psychopathology. With the exception of one study finding increased prenatal maternal NR3C1 methylation in the context of maternal prenatal anxiety as associated to subsequent child behavioral problems (25), we found no other studies that looked at this question in relation to maternal trauma-related psychopathology specifically.

We thus wanted to see if we could replicate and extend the Aswegen et al. (16) study findings. We wanted to do this by: (a) focusing on whether- in the context of maternal IPV-PTSD- maternal NR3C1 promoter region methylation would correlate with child methylation, and (b) investigating whether maternal NR3C1 promoter region methylation during an early sensitive, developmental period for emotion regulation in children would predict child internalizing and externalizing problems at school-age using prospective longitudinal data. Preschool age has been noted to be an important early sensitive period for emotion regulation and related brain development within the context of the mother-child relationship (26, 27). Moreover, psychopathology noted in later childhood and adolescence has been linked to relational difficulties and emotion regulation difficulties during infancy and preschool periods (28–30).

We hypothesized the following in the present study:

- Maternal and child methylation of the NR3C1 gene promoter region would significantly correlate with each other during early childhood,
- Maternal methylation of the NR3C1 gene promoter region during early childhood would negatively and significantly correlate with internalizing (i.e., anxious and depressed) and externalizing (i.e., aggressive and impulsive) symptoms and behavior at school age among children of mothers with IPV-PTSD.

## Materials and methods

### Procedure

The data of the present paper stem from the first and second phase of Geneva Early Childhood Stress conducted between 2010 and 2015 when children were ages 12–42 months and 5–9 years old, respectively. Flyers were posted at different locations, including the Geneva University Hospitals, faculties of medicine, psychology and social sciences, domestic violence agencies and shelters, community centers, daycares, and supermarket bulletin boards. Mothers exposed to domestic violence were oversampled for fear that otherwise, there would not be enough participants with IPV-PTSD—ironically, the inverse was the case and the study recruited many more traumatized mothers than non-traumatized. The procedure has been described in detail elsewhere (31). In short, the protocol included several visits, including one during which mothers completed questionnaires mostly about themselves and one during which they came with their child for the filming of mother child interactions and the acquisition of biological samples including repeated saliva swabs of both the mother and the child using Sarstedt Salivettes^®^ (Salivette^®^, Sarstedt Inc., Rommelsdorf, Germany; www.sarstedt.com).

The Institutional Ethics' Committee of the Geneva University Hospitals gave its approval to the protocol prior to the study, which itself is in accordance with the Helsinki Declaration (32). All adult participants gave written consent after being appropriately informed, mothers gave consent for their child.

### Participants

Inclusion criteria included the following: mothers needing to speak French, consenting to their child's participation in the study and being the biological mothers of their child. Children were needing to be between 12 and 42 months of age. Mothers with active substance abuse or psychotic disorder were excluded; and mothers or children with a handicapping physical or mental problem precluding performance of required study tasks were also excluded. A total of 84 mother-child dyads participated in Phase I. For 48 mothers and children (25 girls, 23 boys, mean age = 27.8 months, SD = 8.8) we had complete data for the measures included in the study as well as sufficient quantity and quality of biological samples for methylation analysis. Of these dyads, 26 had mothers who experienced significant symptoms of IPV-PTSD and 22 had not. These groups did not differ significantly on child age, or sex. However, mothers in the IPV-PTSD group had also experienced more abuse during their own childhood than controls (*p* = 0.024). Nineteen of these dyads did not participate in phase II; but, an additional 7 dyads that had maternal but no child methylation data, did participate in phase II and consequently did provide child behavioral outcome data.

### Measures

Lifetime maternal IPV-PTSD symptoms were assessed using the Clinician Administered PTSD Scale or “CAPS” for the DSM-IV-R (33). The CAPS includes 30 items, which correspond to the DSM-IV diagnosis for PTSD, and yields a total symptom severity score. It is widely used for PTSD assessment, and is characterized by high sensitivity (90%) and specificity (95%), as well as a Cronbach's alpha coefficient of 0.97 (34).

Additionally, physical and sexual abuse during the mothers childhood were measured using the Brief Physical and Sexual Abuse Questionnaire [BPSAQ; (35)] and the Traumatic Life Events Questionnaire [TLEQ; (36)] to supplement questions on traumatic life events not covered by the BPSAQ (37). The TLEQ assesses 22 life events that could fulfill the “A-Criterion” for the DSM-IV diagnosis. The TLEQ shows stability and convergent validity across various studies and minority populations (36). Twelve items that asked about the same type of childhood events as the BPSAQ were eliminated from the TLEQ. Scoring of the BPSAQ was undertaken as described in a previous paper by the first author (38).

Parenting Stress was measured *via* the Parenting Stress Index-Short Form (39). This score includes items related to distress that parents feel in relation to their role as a parent and in light of other personal stressors, as well as parent–child relationship dysfunction, and child behavior that poses difficulty to parents. The PSI-SF has 36 items and each item is assessed on a five-point Likert scale, from 1 (strongly disagree) to 5 (strongly agree). It is a standardized instrument with a validated French translation. The PSI-SF shows high internal consistency (Cronbach's alpha 0.92) (39).

The Child Behavior Checklist [CBCL; (40)] and its subscales were used to measure child psychopathology during Phase II of the study when children were 5–9 years of age (40). The CBCL (6–18-year-old version) is a well-validated parent-report questionnaire that provides a total problems score as well as composite scores for externalizing items encompassing aggressive behavior and internalizing items encompassing anxiety-depressive symptoms. The CBCL shows convergent validity with clinical interviews that take into account child and parental report at the child ages considered (41).

### NR3C1 methylation status

We assessed the methylation levels at 13 CpG sites in NR3C1 exon 1_F_ using bisulfite pyrosequencing, as previously described (10). Briefly, salivary extracted DNA samples in mothers and their children belonging to IPV-PTSD and control groups were bisulfite-converted then PCR amplified using NR3C1 oligonucleotides NR3HumF: 5′-TTTGAAGTTTTTTTAGAGGG-3′ and NR3HumR: 5′-biotin-7-CCCCCAACTCCCCAAAAA-3′. The 403 bp fragment amplicons were pyro-sequenced using the sequencing primer NR3HumS1: 5′-GAGTGGGTTTGGAGT-3′. The Pyro Q-CpG Software determined then automatically the degree of methylation at each CpG site using the C over T pics intensities at the 13 successive CpG sites. **Figure 2** shows the original sequence of the bisulfite-converted and pyro-sequenced region with the 13 successive numerated CpG sites. Supplementary Figures 1, 2 shows the levels of methylation by group for mothers and children.

### Data analysis

We initially performed ordinal logistic regression using the R-based Jamovi Graphic User Interface (version 2.2.5.0). We performed a primary model with two additional models to test for the impact of potential confounders. The initial model used child methylation of the NR3C1 gene across CpGs as the dependent variable; while maternal IPV-PTSD group and maternal average methylation as well as their interaction served as independent variables. Average child and maternal methylation were converted to ranks for analyses, as Shapiro-Wilks tests indicated that they were not normally distributed. A second model further controlled for child sex and age; and, a third model additionally controlled for whether or not mothers reported having experienced abuse (physical or sexual) during their own childhood. To indicate whether results were only reliable using non-parametric methods, we also repeated these models with multiple linear regression and without any transformation. We also performed *post-hoc* power analysis as described in Supplementary material.

Initial omnibus regression analysis was then followed by *post-hoc* tests looking at the individual CpGs with a special focus on those that, according to the available literature, had provided pertinent results in a previous publication that was focused on the mothers (10). Specifically, we investigated the correlations of maternal and child methylation for each group and each CpG.

To test the second hypothesis, we performed regression analyses between maternal NR3C1 methylation at phase I and report of child symptoms during Phase II for overall CBCL as well as the internalizing and externalizing subscales. Given that this combination of phase I and phase II data led to a different subsample, Shapiro-Wilks tests were repeated. Spearman correlations were used if the Shapiro-Wilks tests indicated non-normality.

## Results

### In the context of maternal IPV-PTSD, does maternal methylation of the NR3C1 gene promoter region correlate with child NR3C1 gene methylation?

Logistic regression models included child NR3C1 methylation as the dependent variable along with three independent variables: maternal NR3C1 methylation, PTSD group status, and their interaction. These analyses indicated that all three of these independent variables were significantly predictive (Group: *Z* = 2.22, *p* = 0.026, Maternal NR3C1: *Z* = 3.64, *p* < 0.001, interaction: *Z* = −2.66, *p* = 0.008). These findings remained significant when controlling for child age, sex and maternal abuse history during childhood as potential confounders in models 2 and 3 (see Table 1).

**Table 1:** Ordinal logistic regression models for child methylation of the NR3C1 gene across CpGs as the dependent variable.

**Maternal** **variables**	**Model 1**	**Model 2**	**Model 3**
PTSD group	OR = 10.6 (1.4–92.5) *Z* = 2.22 *p* =0.026	OR = 11.0 (1.3–101) *Z* = 2.20 *p* =0.028	OR = 11.1 (1.3–103) *Z* = 2.19 *p* =0.028
Maternal NR3C1 Methylation	OR = 1.11 (1.05–1.17) *Z* = 3.64 *p* < 0.001	OR = 1.11 (1.05–1.18) *Z* = 3.66 *p* < 0.001	OR = 1.11 (1.05–1.18) *Z* = 3.62 *p* < 0.001
PTSD group ^x^ Maternal NR3C1 Methylation	OR = 0.89 (0.83–0.97) *Z* = −2.66 *p* = 0.008	OR = 0.89 (0.82–0.97) *Z* = −2.64 *p* = 0.008	OR = 0.89 (0.82–0.97) *Z* = −2.61 *p* = 0.009

Model 1, maternal IPV-PTSD group and maternal average methylation as well as their interaction were independent variables. Model 2 controlled for child sex and age, and Model 3 additionally controlled for whether or not mothers reported having experienced abuse (physical or sexual) during their own childhood.

**Figure 1:**
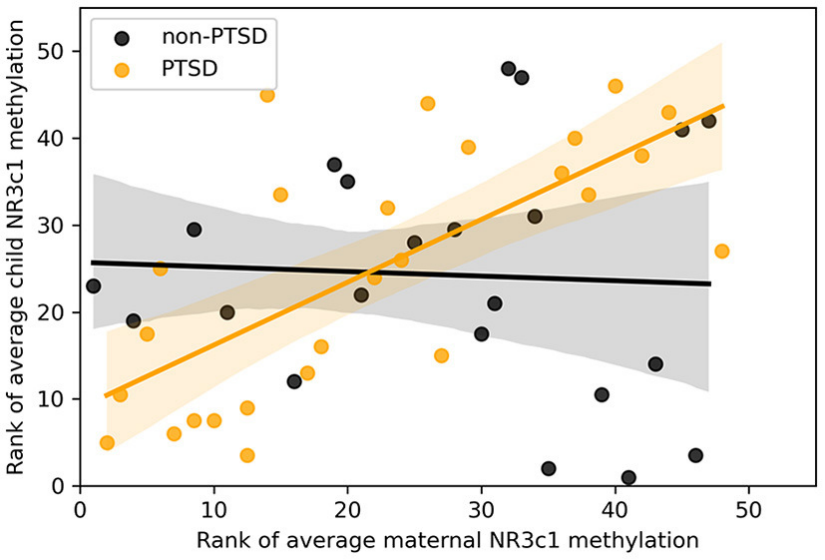
Scatter plot of rank of average methylation of the NR3C1 gene for children and mothers across all 13 CpGs, split by whether mothers had significant symptoms of interpersonal violence related post-traumatic stress disorder.

Reliability analysis using parametric models indicated that, among the independent variables, both maternal NR3C1 alone and the interaction effect of maternal NR3C1 and group remained significant in all models tested (Model 1: Group: *β* = −0.16, *p* = 0.14, Maternal NR3C1: *β* = 0.63, *p* < 0.001, interaction: *β* = −0.57, *p* = 0.043), while the variable PTSD-group by itself was no longer significant. *Post-hoc* Spearman correlations indicated that interaction was significant because maternal NR3C1 correlated with child NR3C1 methylation among mothers in the PTSD group, but not among mothers in the non-PTSD group (see Figure 1, Spearman correlations overall: rs = 0.36, *p* = 0.014, PTSD: rs = 0.71, *p* < 0.001, non-PTSD: rs = −0.08, *p* = 0.73). All together, these findings show that in the context of maternal PTSD, maternal methylation of the NR3C1 gene promoter region predicted child NR3C1 gene methylation.

**Figure 2:**
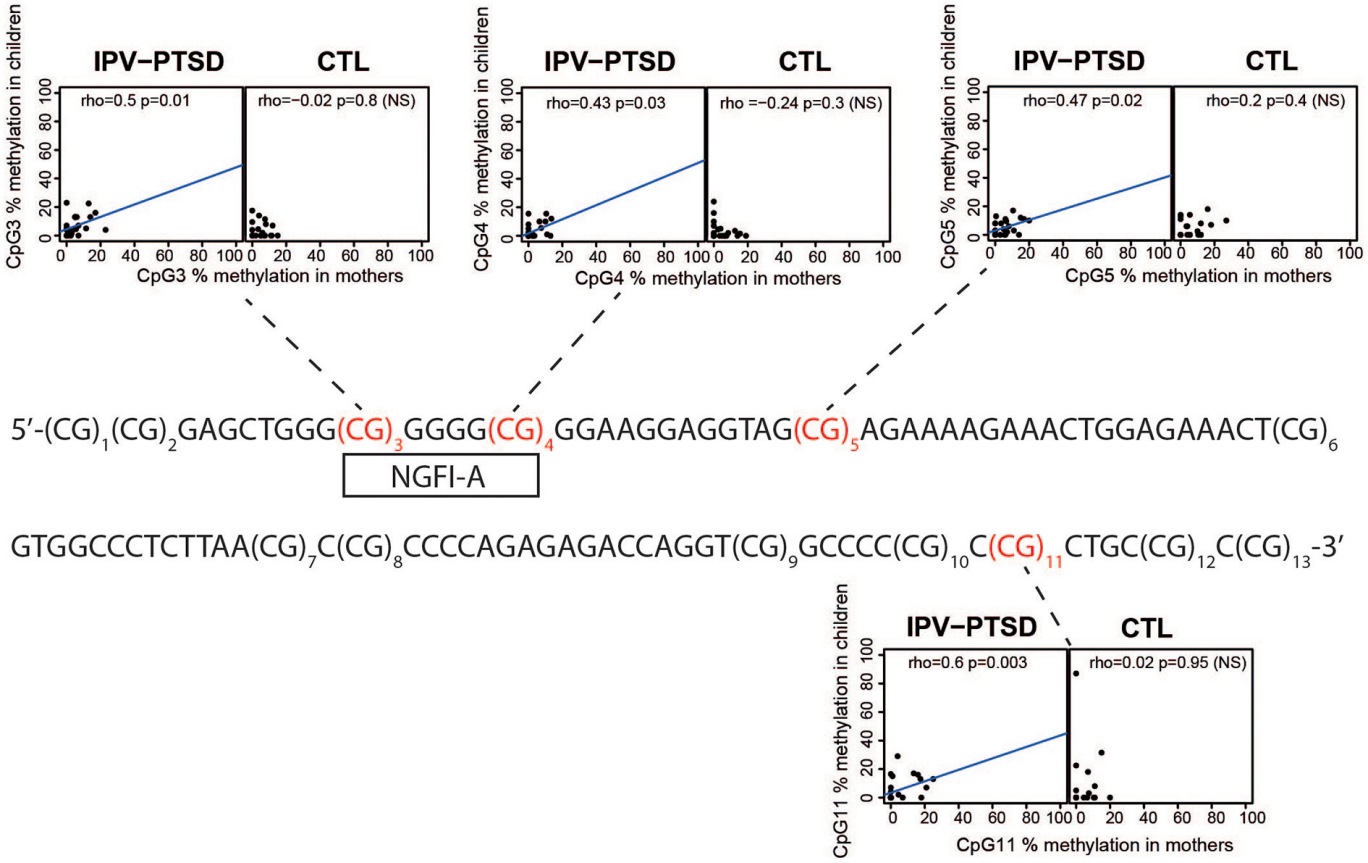
The original sequence of the bisulfite-converted and pyro-sequenced region with the 13 successive numerated CpG sites and scatter plots of CpG methylation in CpGs where there were previous findings linking methylation and IPV-PTSD. These scatter plots show CpG methylation in mothers and children separated by interpersonal violence related post-traumatic stress disorder (IPV-PTSD) or non-PTSD (CTL) among mothers.

Individual sites tested for correlation are shown in Figure 2. We analyzed first the normality of data distribution at the CpG sites using the Shapiro–Wilk test (R function “Shapiro.test”). Then, using non-parametric correlations of CpG methylation in mothers and their children, we tested each site of interest [CpG3, CpG4, CpG5, and CpG11, see Supplemental material in Schechter et al. (10)], using the Spearman's rank correlation (R function “cor.test” with method = “Spearman”). Graphics produced in R with the full possible methylation ranges (0–100%) showed a blue line resulting from a linear model fit only when a correlation is present (Figure 2). The significance of the correlation between the methylation of CpG11 among mothers and that of children in the maternal IPV-PTSD group survived the Bonferroni correction for multiple comparisons (*p* < 0.0125).

### Does maternal NR3C1 promoter region methylation during the early sensitive developmental period for emotion regulation in children predict child internalizing and externalizing problems at school-age?

We tested whether methylation of the NR3C1 promoter region during early sensitive periods of development would be negatively and significantly associated with increased internalizing and externalizing behavior in children later on at school age. To do so, we examined the relationship of maternal NR3C1 methylation to child behavior Phase II of the study when children were 5–9-years-old (7.5 years, SD 0.95). In order to maximize the sample size, we decided not to restrain the sample to include only mothers' NR3C1 methylation whose children had sufficient quantities of salivary DNA for child methylation. As a result, 36 mothers' NR3C1 percentage of overall methylation used as an independent measure to predict child symptoms and behavior as reported by mothers 4–5 years later. We performed the Shapiro-Wilk test for normality within this subsample and found a normal distribution for maternal methylation and CBCL Total problems and Externalizing problems score (Shapiro-Wilk W NS) and a non-normal distribution for CBCL Internalizing problems score (Shapiro-Wilk W *p* = 0.007). Maternal overall NR3C1 methylation was negatively and significantly correlated with CBCL Total Problems score (*r* = −0.359; *p* = 0.031) which was largely accounted for by the moderate correlation to Externalizing Behaviors (*r* = −0.376; *p* = 0.0.024). Internalizing (i.e., anxiety and mood) symptoms were not significantly correlated (Spearman correlation, rs = −0.197, *p* = 0.259) at this level of analysis. We were finally able to show that externalizing behavior was significantly associated not only to maternal NR3C1 methylation, but also to parenting stress during early childhood (*r* = −0.402, *p* = 0.017).

## Discussion

We have presented findings from the prospective, longitudinal study of a rare cohort of mothers and their very young children (ages 12–42 months at baseline) during a sensitive period for the development of emotion and arousal regulation. These mothers share adult histories of interpersonal violence, as well as, for the majority of mothers, childhood physical, sexual abuse and/or family violence exposure, and subsequent related PTSD. We compared these traumatized mothers to a parallel group of mothers and children of the same age, the mothers of whom may or may not have experienced interpersonal violence, but did not have clinically significant PTSD [for more details of the cohort, see Schechter et al. (10)].

Within this particular cohort of mothers and children, among the many multimodal data points collected (10, 31, 42), we have examined the relationship of maternal and child DNA extracted from saliva for methylation of stress-related gene promoter regions including NR3C1 as we discuss here. This paper has examined the relationship of NR3C1 Exon 1F Promoter region methylation between mothers and children, by comparing mothers with vs. without IPV-PTSD and their children during this sensitive period in early childhood. We additionally examined how maternal methylation of this gene during this early period of formative development might independently relate to child psychiatric symptoms and behaviors during school-age. This is the first paper in the literature to do so to our knowledge.

We found that the correlations between mothers and their children of NR3C1 methylation levels overall and at all individual CpG sites of interest were significant only within the IPV-PTSD group. The latter findings support that NR3C1 methylation in mothers is positively and significantly correlated with NR3C1 methylation in their children only in presence of IPV-PTSD in the mothers. Our findings thus replicate and extend those of the recent paper by Van Aswegen et al. (16). The latter study had demonstrated that maternal and child NR3C1 methylation patterns correlated and supported that child and maternal methylation levels with respect to NR3C1 promoter Exon 1_F_ region covary within a sample of 160 typical mothers and children ages 8–16 years. Our present study albeit with a smaller and clinical subsample has indeed similarly demonstrated the significant correlation of maternal and in this instance early childhood NR3C1 methylation yet with the extension of the added dimension of IPV-PTSD.

Our study furthermore supports that a lower percentage of methylation of the NR3C1 promoter region (signifying greater production of the glucocorticoid receptor protein and thus less circulating cortisol), as a “signature” of complex, chronic IPV-PTSD in mothers is related to child relationship problems as marked by parenting stress, and child externalizing behaviors. We previously showed that children of IPV-PTSD mothers showed a blunted cortisol response to a laboratory stressor (14). All together, these findings echoes two previous studies in which childhood adversity in the presence of lower cortisol reactivity moderated the effects of that adversity (i.e., childhood physical abuse) on greater externalizing behaviors (43). Moreover, greater externalizing behavior during early and middle childhood in the wake of parental interpersonal violence has been associated significantly to greater risk for intergenerational transmission of enactment of interpersonal violence with a romantic partner during adolescence and young adulthood (44).

Moreover, we have learned that the latter effects among children have persisted on prospective, longitudinal follow-up and that lower cortisol levels are associated with greater child-self report of peer aggression at ages 5–9 years [Schechter et al., (45), 40th annual meeting of the Swiss Society of Biological Psychiatry (SSBP)] (46). One previous study however has suggested a link by looking directly in the post-mortem human brain (47): the NR3C1 gene promoter region showed decreased methylation among the brains of suicide victims with a history of childhood abuse compared with those of controls (victims of sudden, accidental death with no history of abuse). McGowan et al. (47) noted that, in sum, their findings were particularly relevant, as pituitary ACTH directly reflects central activation of the HPA stress response and hippocampal glucocorticoid receptor activation dampens HPA activity.

One limitation of the present study is indeed the size of the sample that precluded analyses including confounding variables, for example as to gender differences in externalizing vs. internalizing symptoms that have been identified as noted above in studies with larger samples. One factor that affects the ability to obtain complete data on all participants in our study was the necessity of reliance on salivary derived DNA extraction for the methylation analyses. DNA extractions from the child saliva was often insufficient despite our best efforts. We decided in any case to examine the intergenerational aspect of maternal to child behavior through focusing solely on maternal NR3C1 methylation.

Clinical implications of this paper include the use of NR3C1 promoter region 1F methylation as a biomarker to identify children at higher risk of psychopathology. In addition, further research could explore whether modulation of epigenetic targets such as the methylation of the NR3C1 promoter region would influence the orientation toward more personalized mental health care that is more specifically adapted to the relevant endophenotypes (48). Male offspring of mothers who experience male-perpetrated aggression and who show enduring signs of stress in both human and rodent models are a) more likely to display low cortisol baseline and reactivity (and therefore likely greater CG receptor methylation); and b) more likely to express aggressive behavior (14, 46). It is these offspring who are likely to be at greater risk for the perpetuation of intergenerational transmission of violence and related psychopathology; and greater CG receptor methylation (14, 46, 49–51).

## Data availability statement

The raw data supporting the conclusions of this article will be made available by the authors, without undue reservation.

## Ethics statement

The studies involving human participants were reviewed and approved by Commission éthique des Hôpitaux Universitaires de Genève, later known as: Commission cantonale d'éthique de la recherche. Written informed consent to participate in this study was provided by the participants' legal guardian/next of kin.

## Author contributions

MC performed initial analyses, initiated the paper, and was involved in writing of methods and discussion as well as overall editing. LS performed final analyses involving the methylation and having been actively involved in the initial sample processing and epigenetic analyses under supervision of AP-G. LS and AP-G contributed to the writing of all sections of the paper. DM created the data analysis plan, checked all statistical analyses, worked on the main and supplementary figures, and editing. DS and SR as PI and co-PI designed the experimental protocol, were involved at all levels of the prospective, and longitudinal study the data from which this study analyzed and interpreted. DS was furthermore involved in the writing and editing of all sections of this paper. All authors contributed to the article and approved the submitted version.

## Funding

This research was supported by the National Center of Competence in Research (NCCR) SYNAPSY—The Synaptic Bases of Mental Diseases financed by the Swiss National Science Foundation (No. 51AU40_125759).

## Conflict of interest

The authors declare that the research was conducted in the absence of any commercial or financial relationships that could be construed as a potential conflict of interest.

## Publisher's note

All claims expressed in this article are solely those of the authors and do not necessarily represent those of their affiliated organizations, or those of the publisher, the editors and the reviewers. Any product that may be evaluated in this article, or claim that may be made by its manufacturer, is not guaranteed or endorsed by the publisher.
