# Supplementary material for: The relationship of maternal and child methylation of the glucocorticoid receptor NR3C1 during early childhood and subsequent child psychopathology at school-age in the context of maternal interpersonal violence-related post-traumatic stress disorder
Source: Front Psychiatry. 2022 Aug 19;13:919820. doi: 10.3389/fpsyt.2022.919820 (PMC9437341; doi:10.3389/fpsyt.2022.919820)
Supplement: Supplementary file 2 [file Data_Sheet_1.docx]

Supplementary Figures

Figure S1 methylation of maternal CpGs of the NR3C1 gene by group of PTSD group status


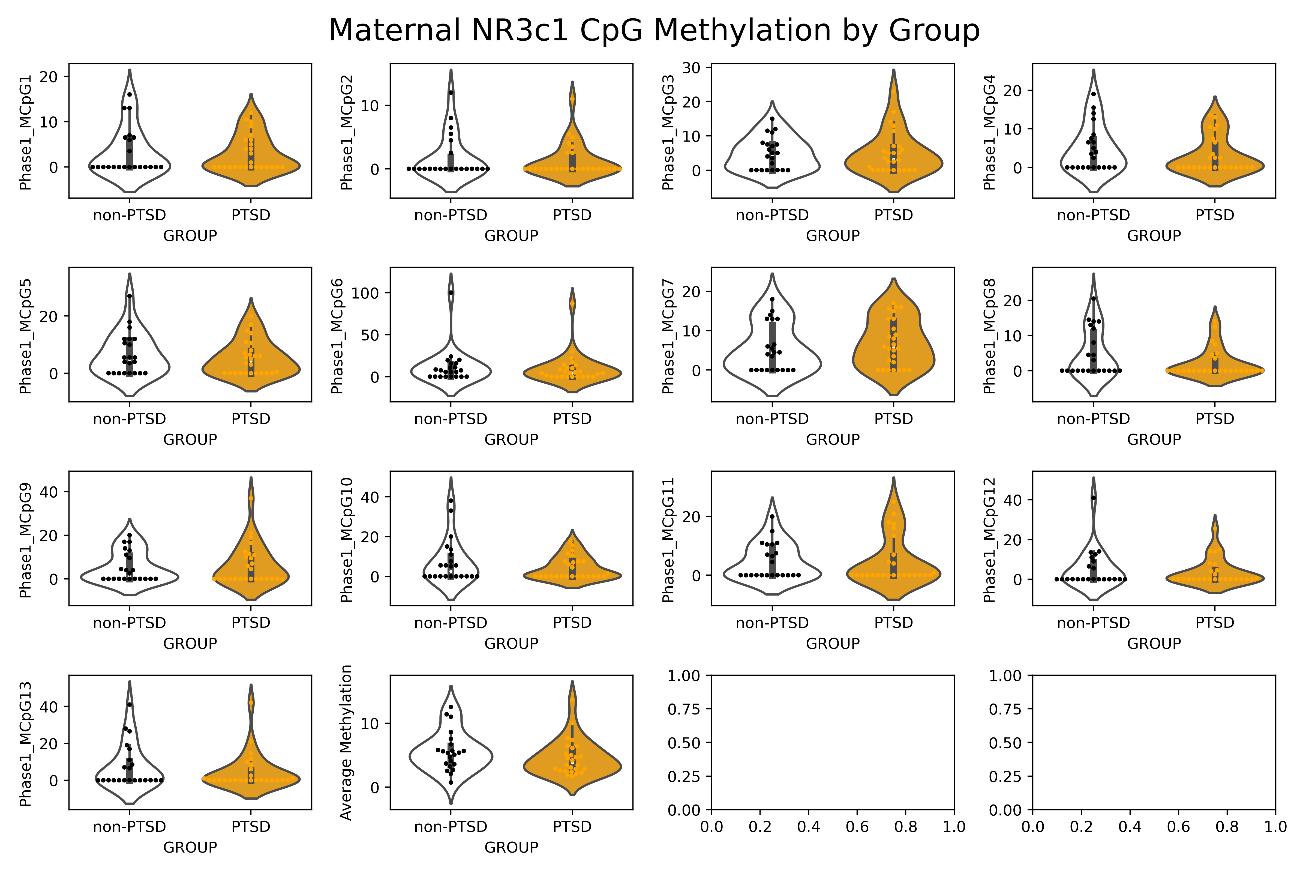


Figure S2 methylation of child CpGs of the NR3C1 gene by group of maternal PTSD group status


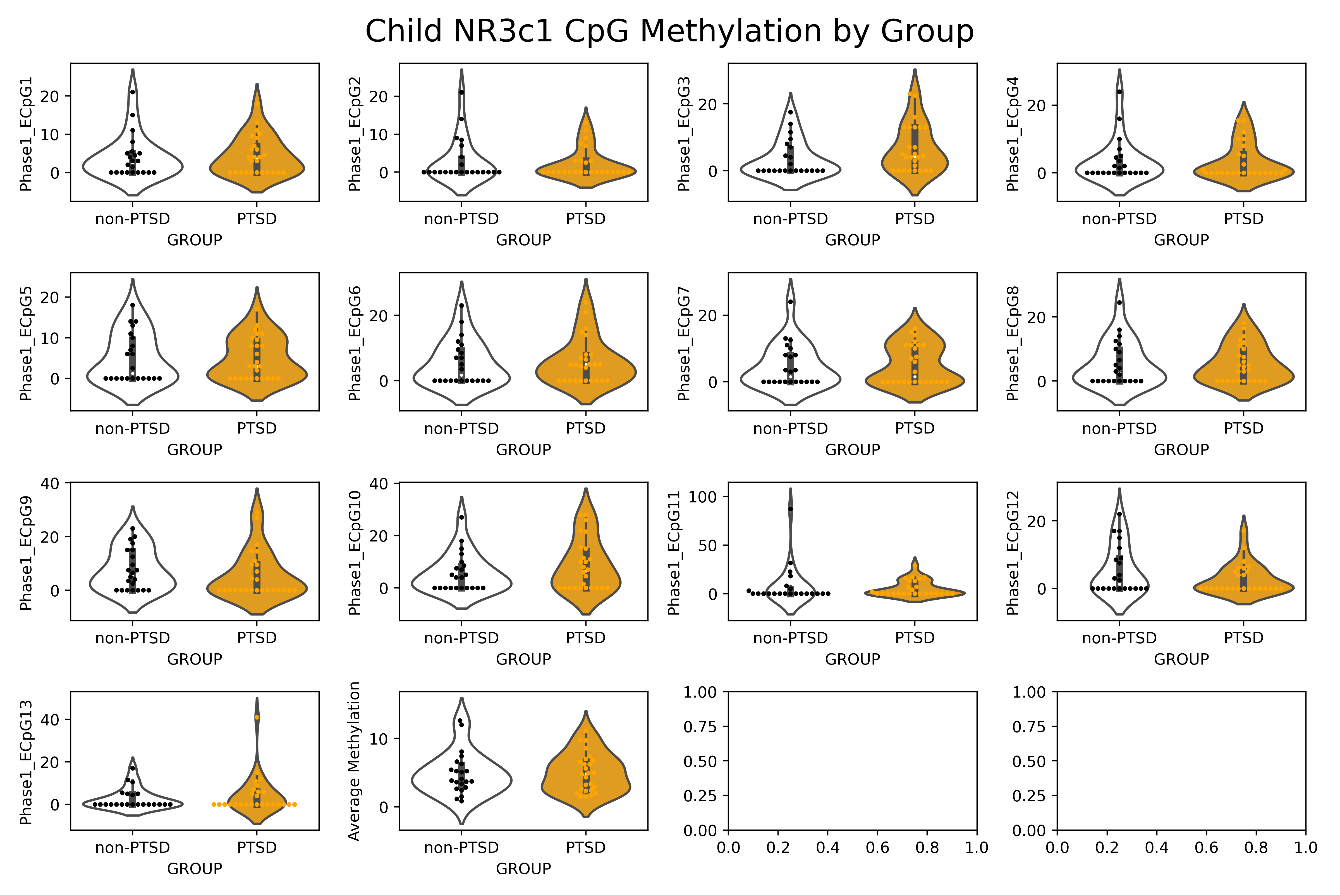


**Power analysis**

In order to retrospectively assess the power of the statistical analysis, we performed a Monte Carlo analysis for linear regression in MATLAB 2017b (MathWorks). We did this after having performed linear regression following transformation of non-dichotomous variables into ranks, and having found fairly similar significances as in the logistic regression. The tested model here had -like the actual model- mean child methylation and maternal group (PTSD or non-PTSD) as well as their interaction as independent variables, and mean child methylation as dependent variable. We performed this in 3 forms with 1000 permutations each. Once with 48 participants (26 PTSD), once with 80 (40 PTSD) and once with 120 (60 PTSD) to test what effect size was necessary at our sample size and how that would be changed at bigger sample sizes. For each of these sample we artificially created 1000 random distributions.

We then designed a grid where we introduced correlations between maternal and child methylation within the PTSD group only in steps of r=0.03 and group differences in child methylation at steps of 0.04 standard deviations. Correlations were introduced using the “*sde_correlate*” function of the SDE master toolbox for MATLAB ^1^. For each group size and each step we calculated mean significance as well as percentage of permutations that would have ended in significant (p<0.05) results.

Results are given in full in the associated excel file and in abbreviated form below (Table S1). They indicate that group differences might be overestimated at lower levels of difference, but not once effect sizes mount. They also indicate that effect sizes had to be fairly large at our sample size (which they were as indicated by a within group correlation of r=0.71 among dyads with maternal PTSD). Still, medium effect sizes (of r=0.54 and 072 standard deviations difference) would likely still have yielded significant results for at least the association of child methylation (89%) and maternal methylation and to a lesser degree also the interaction (50%). Such effect sizes become highly likely significant once sample size gets increased to 80 (99% for maternal mean methylation, 73% for interaction but still only 57% for group). Increasing the sample further to 120 dyads would have additional benefits, but would still not decrease needed effect sizes to very small levels.

|  |  |  | **Effect size** | | | | | | | | | | | | |
| --- | --- | --- | --- | --- | --- | --- | --- | --- | --- | --- | --- | --- | --- | --- | --- |
|  |  | **r** | **0** | **0.06** | **0.12** | **0.18** | **0.24** | **0.3** | **0.36** | **0.42** | **0.48** | **0.54** | **0.6** | **0.66** | **0.72** |
|  |  | **d** | **0** | **0.08** | **0.16** | **0.24** | **0.32** | **0.4** | **0.48** | **0.56** | **0.64** | **0.72** | **0.8** | **0.88** | **0.96** |
| n=48 | Group | Percent significant at p<0.05 | 13.5 | 13.8 | 17.5 | 22.7 | 28.2 | 33 | 38.8 | 46.1 | 52.6 | 57.5 | 60.6 | 62.5 | 62.3 |
|  | Mean Maternal NR3c1 |  | 5.7 | 6.6 | 9.8 | 13.6 | 20.4 | 29.2 | 41.1 | 59 | 76.1 | 89.8 | 96.6 | 99.1 | 100 |
|  | Group*Mean Maternal NR3c1 |  | 5 | 5.7 | 6.5 | 9.7 | 12.5 | 16.4 | 22.2 | 28.4 | 37.6 | 50.2 | 63.9 | 75.5 | 86.3 |
| n=80 | Group |  | 12 | 13.7 | 15.8 | 20.1 | 25.9 | 33 | 38.8 | 46.1 | 52.6 | 57.5 | 60.6 | 62.5 | 62.3 |
|  | Mean Maternal NR3c1 |  | 5.4 | 6.6 | 13.5 | 25.3 | 44 | 63.2 | 80.1 | 93.9 | 99 | 99.8 | 100 | 100 | 100 |
|  | Group*Mean Maternal NR3c1 |  | 4.4 | 4.8 | 5.4 | 8.2 | 11.8 | 17.3 | 25.7 | 39.2 | 55.8 | 73.2 | 89.3 | 97.3 | 99.6 |
| n=120 | Group |  | 12 | 16.1 | 25.1 | 34 | 46.5 | 58.1 | 67.6 | 78.1 | 86 | 91 | 94.2 | 95.9 | 96.8 |
|  | Mean Maternal NR3c1 |  | 6 | 6.9 | 13.3 | 26.2 | 41 | 58.8 | 75 | 90.3 | 97.6 | 99.7 | 100 | 100 | 100 |
|  | Group*Mean Maternal NR3c1 |  | 5.8 | 7.2 | 11.4 | 17.3 | 24.4 | 34.7 | 46.8 | 59.7 | 77.4 | 88.8 | 95.5 | 99.6 | 99.9 |

Table S1: Power analysis with mean percentage of permutations significant dependent on effect size. D indicates difference between groups in standard deviations.

1. *SDETools: A Matlab Toolbox for the Numerical Solution of Stochastic Differential Equations (SDEs).* [*https://github.com/horchler/SDETools*](https://github.com/horchler/SDETools) [computer program]. github2017.
